# Miocene Diversification and High-Altitude Adaptation of *Parnassius* Butterflies (Lepidoptera: Papilionidae) in Qinghai–Tibet Plateau Revealed by Large-Scale Transcriptomic Data

**DOI:** 10.3390/insects11110754

**Published:** 2020-11-03

**Authors:** Chengyong Su, Tingting Xie, Yunliang Wang, Chengcai Si, Luyan Li, Junye Ma, Chunxiang Li, Xiaoyan Sun, Jiasheng Hao, Qun Yang

**Affiliations:** 1 College of Life Sciences, Anhui Normal University, Wuhu 241000, China; sky475342@163.com (C.S.); xie517147151@outlook.com (T.X.); wyl1119@163.com (Y.W.); sichengcai2017@163.com (C.S.); 2 State Key Laboratory of Palaeobiology and Stratigraphy, Center for Excellence in Life and Palaeoenvironment, Nanjing Institute of Geology and Paleontology, Chinese Academy of Sciences, Nanjing 210008, China; lyli@nigpas.ac.cn (L.L.); jyma@nigpas.ac.cn (J.M.); cxli@nigpas.ac.cn (C.L.); xysun@nigpas.ac.cn (X.S.); 3 Xuzhou Engineering Research Center of Biochemical Resources Efficient Utilization, Xuzhou College of Industrial Technology, Xuzhou 221140, China; 4 College of Earth & Planetary Sciences, University of Chinese Academy of Sciences, Beijing 100049, China

**Keywords:** accelerated diversification, conservation, divergence time, high-altitude adaptation, *Parnassius* butterflies, phylogeny, positive selection, Qinghai–Tibet Plateau, transcriptome

## Abstract

**Simple Summary:**

*Parnassius* butterflies have contributed to fundamental studies in biogeography, insect–plant interactions, and other fields of conservation biology and ecology. However, the early evolutionary pattern and molecular adaptation mechanism of this alpine butterfly group to high altitudes in Qinghai–Tibet Plateau are poorly understood up to now. In this study, we report for the first time, a relatively large-scale transcriptomic dataset of eight *Parnassius* species and their two closely related papilionid species, a dated phylogeny based on hundreds of gene sequences, and potential genetic mechanisms underlying the high-altitude adaptation by investigating changes in evolutionary rates and positively selected genes. Overall, our findings indicate that the transcriptome data sets reported here can provide some new insights into the spatiotemporally evolutionary pattern and high altitude adaptation of *Parnassius* butterflies from the extrinsic and intrinsic view, and will support further expressional and functional studies that will help interested researchers to address evolution, biodiversity and conservation questions concerning *Parnassius* and other butterfly species.

**Abstract:**

The early evolutionary pattern and molecular adaptation mechanism of alpine *Parnassius* butterflies to high altitudes in Qinghai–Tibet Plateau are poorly understood up to now, due to difficulties in sampling, limited sequence data, and time calibration issues. Here, we present large-scale transcriptomic datasets of eight representative *Parnassius* species to reveal the phylogenetic timescale and potential genetic basis for high-altitude adaptation with multiple analytic strategies using 476 orthologous genes. Our phylogenetic results strongly supported that the subgenus *Parnassius* formed a well-resolved basal clade, and the subgenera *Tadumia* and *Kailasius* were closely related in the phylogenetic trees. In addition, molecular dating analyses showed that the *Parnassius* began to diverge at about 13.0 to 14.3 million years ago (middle Miocene), correlated with their hostplant’s spatiotemporal distributions, as well as geological and palaeoenvironmental changes of the Qinghai–Tibet Plateau. Moreover, the accelerated evolutionary rate, candidate positively selected genes and their potentially functional changes were detected, probably contributed to the high-altitude adaptation of *Parnassius* species. Overall, our study provided some new insights into the spatiotemporally evolutionary pattern and high altitude adaptation of *Parnassius* butterflies from the extrinsic and intrinsic view, which will help to address evolution, biodiversity, and conservation questions concerning *Parnassius* and other butterfly species.

## 1. Introduction

The Qinghai–Tibet Plateau (QTP), as the “Third Polar” of the world, offers extreme and substantially variable environments with low level of oxygen, low temperature and strong ultraviolet radiation, making it ideal for studying the high-altitude adaptation and species radiation of organisms [1,2]. Previous studies have shown that the uplifts of QTP and its adjacent areas not only provide unique conditions for the adaptive evolution of many animal and plant groups [3,4,5], but also have greatly affected the spatiotemporally evolutionary patterns of organisms in Eurasia [6,7,8]. For example, radiations of groups of plants are shown to be triggered by extensive uplifts of the QTP [3,5], the *Melitaea* butterflies have been reported to diversify at significantly varying rates, contemporary with the QTP orogeny and climatic changes [6], the phylogeographic pattern of Eurasian passerines was associated with the Himalayan–Tibetan uplift [7].

Comparative analysis revealed that animals in high-altitude environments can evolve morphological, physiological and genetic adaptations to counter harsh environmental challenges, such as altered body masses, enhanced oxygen delivery, as well as metabolic and genetic changes [1,9,10]. For insects, previous research mainly focused on individual species under short-term artificial stressor treatment (e.g., hypoxia or normoxia, low, or high temperature) [1,9,11]. However, the long-term evolutionary adaptations to high altitudes among closely related species are not fully addressed. In this study, we chose to sample native butterfly species and use large scale transcriptomic data to identify potential genetic mechanisms for the high-altitude adaptations.

The genus *Parnassius* is a typical mountainous butterfly group, mainly distributed along a broad elevational range of 3000–5000 m on QTP and its adjacent slopes. Previous studies suggest that the *Parnassius* ancestors probably originated in regions of Central Asia to Western China [12,13,14], and their rapid radiations were likely related with the QTP uplift; a few species subsequently colonized at low-altitude habitats, such as *Parnassius glacialis* distributed in central to eastern China around 200–1000 m altitudes, suggesting a unique evolutionary history in Parnassiinae [12,13,14]. *Parnassius* butterflies have contributed to fundamental studies in biogeography, insect–plant interactions, and other fields of conservation biology and ecology, suitable for illustrating how modern biotas have been shaped by past geological and climatic events, and improving our ability to predict and mitigate the threats to alpine species posed by global warming and environmental disruption.

Previous studies on phylogenetic relationships of parnassian butterflies have been mainly based on a limited number of sequence markers, and the phylogenetic backbone of this group has not been resolved [12,13,14,15]. Here we use high-throughput sequencing technologies, especially RNA sequencing (RNA-Seq) [16] to massively collect the gene sequences at the genomic level to explore phylogeny, spatiotemporal diversification, and adaptive molecular evolution. We report for the first time, a relatively large-scale transcriptomic dataset of eight *Parnassius* species and their two closely related papilionid species, a dated phylogeny based on hundreds of gene sequences, and potential genetic mechanisms underlying the high-altitude adaptation by investigating changes in evolutionary rates and positively selected genes (PSGs).

## 2. Materials and Methods

### 2.1. Sample Collection

For de novo transcriptome sequencing, we sampled eight *Parnassius* species representing five subgenera and other two Papilionidae species (*Sericinus montelus* and *Papilio polytes*) as the outgroup (Supplementary Table S1). Among the eight *Parnassius* species, seven are high-altitude (HA) (*Parnassius epaphus*, *Parnassius jacquemontii*, *Parnassius cephalus*, *Parnassius imperator*, *Parnassius acdestis*, *Parnassius simo*, and *Parnassius orleans*), and one is low-altitude (LA) species (*Parnassius glacialis*) (Supplementary Table S1). Specimens were initially preserved in RNA stabilization solution (Sangon Biotech, Shanghai, China) in the field and transferred to −80 °C until RNA extraction. Flight muscle tissues from the thorax of one to three adult samples per species were used for purified RNA extraction.

### 2.2. mRNA-Seq Library Construction, Illumina Sequencing, Assembly, and Annotation

Libraries construction, Illumina sequencing and assembly were performed following the methods in previous study [17]. Total RNA was extracted using Trizol Reagent (Invitrogen, Carlsbad, CA, USA) according to the manufacturer’s instructions. Quality and integrity of RNA were determined using a NanoDrop spectrophotometer (Thermo Scientific, Barrington, IL, USA) and Agilent 2100 Bioanalyzer (Agilent Technologies, Palo Alto, CA, USA). The sequencing library was paired-end sequenced using the Illumina NextSeq 500 platform (Shanghai Personal Biotechnology Co. Ltd., Shanghai, China). After the adaptor contamination was removed, the reads were screened from the 3′ to 5′ end to trim the bases with a quality score of Q < 20 by using 5-bp windows, and the reads with final length of less than 50 bp and ambiguous nucleotides were removed. After quality filtering, the quality reads per specimen were collected and then de novo assembled into contigs and transcripts using Trinity v2.0.2 (https://github.com/trinityrnaseq/trinityrnaseq/releases, accessed on 28 February 2019). The sequencing data has been deposited on the GenBank under BioProject number of PRJNA591246.

All the transcripts were searched against the non-redundant protein sequences database (NR) in the National Center for Biotechnology Information (NCBI) by using the Basic Local Alignment Search Tool (BLAST, E-value < 10^−5^, https://blast.ncbi.nlm.nih.gov, accessed on 28, February, 2019), and the top-hit transcripts were selected as unigenes. All unigenes were then searched against SWISS-PROT, Kyoto Encyclopedia of Genes and Genome (KEGG), and non-supervised orthologous groups (eggNOG) database (http://eggnog.embl.de/, accessed on 1 March 2019) for functional category annotation. The Blast2GO program [18] was used to obtain gene ontology (GO) annotation on the basis of GO terms with the BLASTX algorithm (Blast v2.2.28, E-value < 10^−5^), and the KAAS (KEGG Automatic Annotation Server, www.genome.jp/tools/kaas/, accessed on 1 March 2019) with bidirectional best-hit strategy was used to assign KEGG orthology terms (KO) and to identify the pathways involved. If a unigene could not be aligned to any of the above databases, its sequence direction and amino acid sequence prediction were conducted via the Transdecoder in the Trinity program package. Transcriptome assembly completeness was determined by Benchmarking Universal Single-Copy Orthologs (BUSCO, version 4.0.2) based on conserved insect orthologs (https://busco.ezlab.org/, accessed on 3 March 2019) with default settings.

### 2.3. Check for Cross-Contaminations

The CroCo 1.1 was used to check the cross-contamination level in our different sequencing runs [19], which was developed for identifying and removing cross contaminants from assembled transcriptomes. In addition, this method can also be applied to estimate relative coverage for each coding DNA sequence (CDS) of each species.

### 2.4. Ortholog Identification and Alignment

Single-copy orthologous genes of the ten papilionid transcriptome sequences were identified using OrthoMCL [20]. CDS regions of each species were used as queries to search against the other nine species (reciprocal best BLAST hits). The best hits with similarity > 70% and E-value < 10^−6^ were retrieved and only one-to-one orthologous genes existing in all ten species were retained for further analyses. All identified orthologous sequences were aligned using MAFFT v7.017 with the codon option and L-INS-i algorithm [21]. Potentially poor alignments or low quality regions were trimmed using Gblocks v.0.91b with half gaps allowed [22]. After the trimming process, alignments shorter than 100 bp were discarded.

### 2.5. Phylogenetic Analysis

After alignment and trimming, all amino acids and nucleotides of protein-coding genes (PCGs) were concatenated into “supergenes”. *Parnassius* phylogenetic trees were reconstructed using *S. montelus* and *P. polytes* as the outgroups. The best-fit nucleotide model partitioned by three codon positions was selected with PartitionFinder and ModelFinder [23,24], the best-fit amino acid model was selected with ProtTest 3 [25], both following the corrected Akaike information criterion (AICc) (Supplementary Table S2). In addition, the best-fit codon model was also used in resolving complicated phylogenies [26].

Maximum likelihood (ML) and Bayesian inference (BI) methods were used to reconstruct the species-level tree. ML analyses were performed in IQ-TREE v.1.6.1 [27], with the branch support values estimated and single branch tests using the embedded ultrafast bootstrap approach (UFBoot) and SH-like approximate likelihood ratio test (SH-aLRT), respectively [28]. Bayesian analyses were performed in MrBayes 3.2 [26], using two Markov Chain Monte Carlo (MCMC) chains running for 2 million generations with sampling each hundredth generation. Each run had four chains, one cold and three heated. When the convergence of MCMC chains was achieved (the average standard deviation of split frequencies (StdDev) < 0.01, potential scale reduction factor (PSRF) ≈ 1), the first 25% of the sampled generations were discarded as “burn-in”.

### 2.6. Divergence Time Estimation

Divergence dates were estimated with the program BEAST v1.8.3 [29], using both the amino acid and nucleotide data of the ten species under the uncorrelated lognormal relaxed-clock model. The amino acid data were unpartitioned with the best-fit amino acid model (Supplementary Table S2), while the nucleotide data were partitioned into codon positions with independent GTR+G model. The branching process prior was set to birth-death process. 

**Figure 1:**
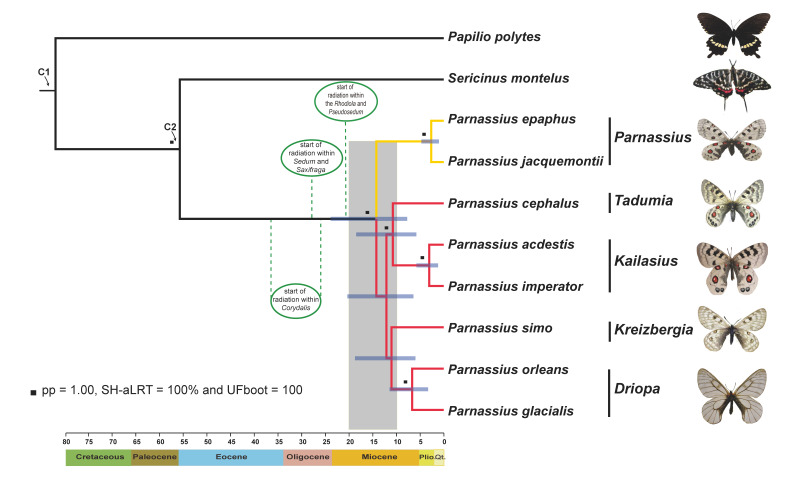
Phylogenetic relationships and divergence time estimates of the genus *Parnassius* based on amino acids of 476 orthologous genes. The tree was inferred under the maximum likelihood and Bayesian analyses. Divergence times were estimated under the birth-death prior shown with the 95% confidence intervals (blue bars); C1 and C2 show the time calibration points; the border in gray shows the time range (10–20 Ma) of the progressive extension of the uplift of the Qinghai–Tibet Plateau (QTP) and related environmental changes. Lineages in yellow and in red mostly feed on the hostplants Crassulaceae + Saxifragaceae (e.g., *Sedum*, *Rhodiola*, *Pseudosedum* and *Saxifraga*) and Papaveraceae (e.g., *Corydalis*), respectively.

We followed a conservative approach which assumes that fossil records only provide a minimum age when setting the priors for calibration points, and used uniform prior distributions for all calibration points [30,31]. Here, we constrained the crown age of Papilionidae (Node C1, Figure 1) with a minimum age of 47.8 million years ago (Ma) based on the oldest swallowtail fossil *Praepapilio colorado* (early Eocene (Lutetian), the Green River formation in Colorado) [32], and that of Parnassiinae (Node C2, Figure 1) with a minimum age of 23.03 Ma (early Miocene) based on the fossil *Thaites ruminiana* from limestone in the Niveau du gypse d’Aix Formation of France [33]. Uniform distributions of two fossil calibrations were maximally bounded at 150 Ma, following the practice by Allio et al. [31].

The calibrated nodes were constrained to be monophyletic as recommended in Drummond and Bouckaert [34]. Three independent MCMC runs of 50 million generations, sampling every 5000 generations were performed. Tracer v1.6 was used to check the effective sample size values to ensure that posterior distributions were sufficiently sampled [35]. Using LogCombiner v. 1.8.3 [29], the posterior distributions of trees from the three runs were combined, discarding the first 10% trees of each run as “burn-in”. TreeAnnotator v.1.8.3 was used to extract the mean and the 95% confidence interval (CI) of the posterior distributions [29].

### 2.7. Evolutionary Rate and Positive Selection Analyses

The codeml program in the PAML package [36] with the free ratio model (model = 1) was used in this study to estimate the evolutionary rate following Yang et al. and Wang et al. [37,38]. After the alignment quality control, only high quality loci without alignment problems were used for analyses. The lineage-specific mean ratios of nonsynonymous (dN) to synonymous (dS) substitutions rates (ω = dN/dS) were estimated for each ortholog, and concatenated all orthologs, using the species-level tree of this study as the guide tree. Parameters, including dN, dS, dN/dS, N × dN, and S × dS values, were obtained for each branch. Genes with N × dN or S × dS < 1 or dS > 2 were filtered following the approach of the previous study [39]. Statistical significance of the differences in the dN/dS ratios along different lineages were conducted using the Wilcoxon rank sum test.

To find genes that potentially experienced positive selection underlying the high-altitude adaptation, the branch-site model (model = 2) in the PAML package was used to detect positively selected genes (PSGs) [36], with the *Parnassius* common ancestor being specified as the foreground branch. To avoid false positive results, the following rigorous criteria were used: The dN/dS ratio (ω) greater than 1 on the foreground branch; the positively selected sites with a posterior probability greater than 0.95 [40]; and the *p*-value ≤ 0.05 in the likelihood ratio test [41]. Finally, the substitution specificity of positively selected sites in the candidate PSGs was recognized with more insect homologous gene sequences in NCBI nr/nt database. Candidate PSGs without identical substitutions at the positively selected sites were used for later analysis.

### 2.8. Protein Three-Dimensional (3D) Structure Simulation

The PSGs’ functional domains were obtained from the PROSITE database (https://prosite.expasy.org, accessed on 20 December 2019). The 3D structures of PSGs were predicated using Phyre2 [42] and the SWISS-MODEL server (https://swissmodel.expasy.org, accessed on 20 December 2019), then evaluated on the SAVES website (https://servicesn.mbi.ucla.edu/SAVES, accessed on 27 December 2019). All the high-confidence 3D structures obtained both on Phyre2 and SWISS-MODEL servers were visualized using PyMOL [43]. The electrostatic potentials at the surfaces of the predicted protein structures were obtained using PyMOL with ABPS and PDB2PQR [44].

## 3. Results

### 3.1. Sequencing, De Novo Assembly, and Annotation

For *Parnassius* species, about 515 million clean reads with 74.75 Gbp in length were obtained from Illumina sequencing, and assembled into more than 361 thousand unigenes with a mean of 493 bp in size (Supplementary Table S3). In total, average of 25,908 (56.87%) unigenes per *Parnassius* sample had significant matches to currently known proteins in databases (Supplementary Table S4). Of all databases, NR had the largest match value (24,810, 54.35%), followed by eggNOG (24,761, 54.40%), SwissProt (17,513, 37.47%) and GO (16,555, 35.43%) databases (Supplementary Table S4). The results of annotation, including NR, GO category distribution, eggNOG functional classification and the KEGG pathway of unigenes were similar (Supplementary Figures S1–S3), suggesting high consistencies of de novo assembly and annotation. Average of 1304 (78.6%) complete and fragmented BUSCOs were identified in *Parnassius* species, while 1449 (87.4%) and 1379 (83.2%) found in *S. montelus* and *P. polytes*, respectively (Supplementary Figure S4). Thus, our results indicate that the 10 transcriptomes were well assembled and relatively complete. The BUSCO analysis showed that our assembled unigenes were better than the majority of other 46 arthropod species in assembly and annotation completeness [45]. The cross-contamination check using CroCo recovered a low level of cross contamination with a mean of 108 out of 10,000 (1.08%) contigs contaminated by species (Supplementary Table S5).

### 3.2. Phylogenetic Analysis

After our alignment treatments and trimming for quality control, 476 single-copy orthologous protein-coding genes were recovered from the determined sequence data, covering 435,231 nucleotides or 145,077 amino acids (Supplementary dataset S1). Nearly all the resultant phylogenetic trees were robust when the best-fit amino acid and codon models were used (posterior probabilities (PP) > 0.95, SH-aLRT ≥ 80% and UFboot ≥ 95; Figure 1 and Supplementary Figure S5). The subgenus *Parnassius,* which use Crassulaceae as their hostplant, formed a basal clade, sister to other subgenera mostly specialized in feeding on the hostplant *Corydalis*, supporting the results of previous analyses [13,14]. The subgenus *Tadumia* was shown to be more closely related to *Kailasius* than to the *Kreizbergia* + *Driopa* clade (Figure 1), consistent with the ML analysis of previous study [13]. The nucleotide model-based ML and Bayesian analyses produced basically the same phylogenies, with some weak node supports as found in previous studies [12,13,14,15,46] (Supplementary Figure S5).

### 3.3. Divergence Time Estimates

Multiple molecular dating analyses using amino acid and nucleotide datasets, yielded similar divergence time estimates (Table 1). Our estimates of evolutionary timescale suggested that the diversification of *Parnassius* initiated about 13.0 to 14.3 Ma, followed by successive splittings during 9.9 to 12.1 Ma (Figure 1 and Table 1, birth-death process), supporting the previous hypotheses of their rapidly adaptive radiations [13,14]. These diversifications are largely coeval with the progressive QTP orogenic events and related environmental changes that occurred around 10–20 Ma [47,48,49], and their hostplants’ rapid diversification from the QTP region to Central Asia (e.g., *Corydalis*, *Rhodiola*, *Pseudosedum*, *Sedum,* and *Saxifraga*) [50,51,52]. Though somewhat different from those of previous studies [12,13,53], our dating estimates are congruent with other studies in recent years [14,31,46].

### 3.4. Accelerated Evolution of the Subgeneric Diversification

**Figure 2:**
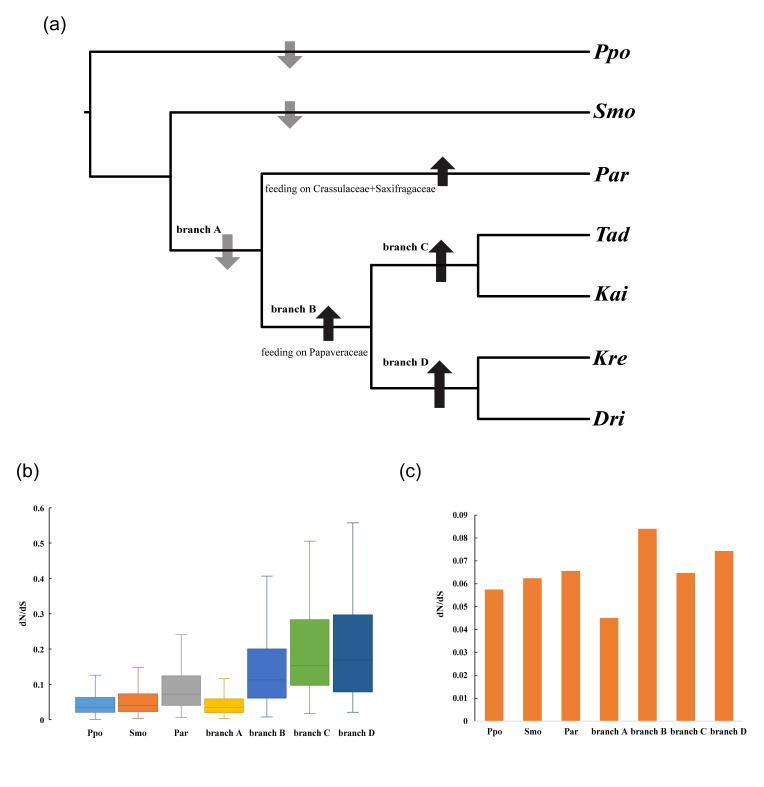
Phylogenetic tree used in this study (**a**) and branch specific ratios of nonsynonymous (dN) to synonymous (dS) substitutions rates (dN/dS ratios) obtained from different data sets (**b**,**c**). Arrows in (**a**) indicate decreased or increased dN/dS ratios. The dN/dS ratios for branches were estimated from each ortholog (**b**) and concatenated all orthologs (**c**). Ppo, *Papilio polytes*; Smo, *Sericinus montelus*; Par, subgenus *Parnassius*; Tad, subgenus *Tadumia*; Kai, subgenus *Kailasius*; Kre, subgenus *Kreizbergia*; Dri, subgenus *Driopa*.

To better understand the evolutionary dynamics of *Parnassius* butterflies, we compared the overall difference in selective constraints in different branches. Each putative single copy orthologous gene was evaluated for evolutionary parameters including dN, dS, and dN/dS, using the free-ratio model in PAML. Across all 476 orthologous genes, the branch of subgenus *Parnassius* (mostly feed on the hostplants Crassulaceae and Saxifragaceae) had a significantly higher ratio of dN/dS than branch Ppo (*Papilio polytes*, Wilcoxon rank sum test, *p* < 6.4 × 10^−19^), branch Smo (*Sericinus montelus*, Wilcoxon rank sum test, *p* < 1.1 × 10^−11^) and branch A (ancestral branch of genus *Parnassius*, Wilcoxon rank sum test, *p* < 7.5 × 10^−24^), while the branch B, C and D (ancestral branches of other subgenera which mostly feed on the hostplant Papaveraceae) all had higher ratios than other branches mentioned above, including the branch of subgenus *Parnassius* (Wilcoxon rank sum test, *p* < 3.0 × 10^−3^, Figure 2a,b). In addition, analysis of concatenated gene sequence was also conducted and showed the same result in general: Ancestral branches of subgenera in *Parnassius* harbored higher dN/dS ratios, suggesting accelerated evolutionary rate for Crassulaceae+Saxifragaceae feeders (subgenus *Parnassius*) and Papaveraceae feeders (all *Parnassius* except subgenus *Parnassius*) (Figure 2c). All above indicated that accelerated evolution occurred after the early splitting event in the genus *Parnassius* and in the process of subgeneric origin and diversification (Figure 2a), which is likely to be caused by their hostplant shifts [14].

### 3.5. Positively Selected Genes

After applying rigorous criteria to identify genes that are likely to be important in high-altitude adaptation for *Parnassius* species, five candidate PSGs with high quality alignments remained on the list (Table 2 and Supplementary Table S6). Moreover, when the substitutions for these five genes were examined with more available insect sequences, no identical substitutions were found in those insects (Supplementary Table S7). Three-dimensional (3D) structure simulations were also conducted to examine the possible effects of these mutations on the protein structure, and fortunately, three candidate PSGs (ILF2, PRPF17, and CDASE) can be aligned to the suitable templates in the PDB data bank to build homology 3D structures.

**Figure 3:**
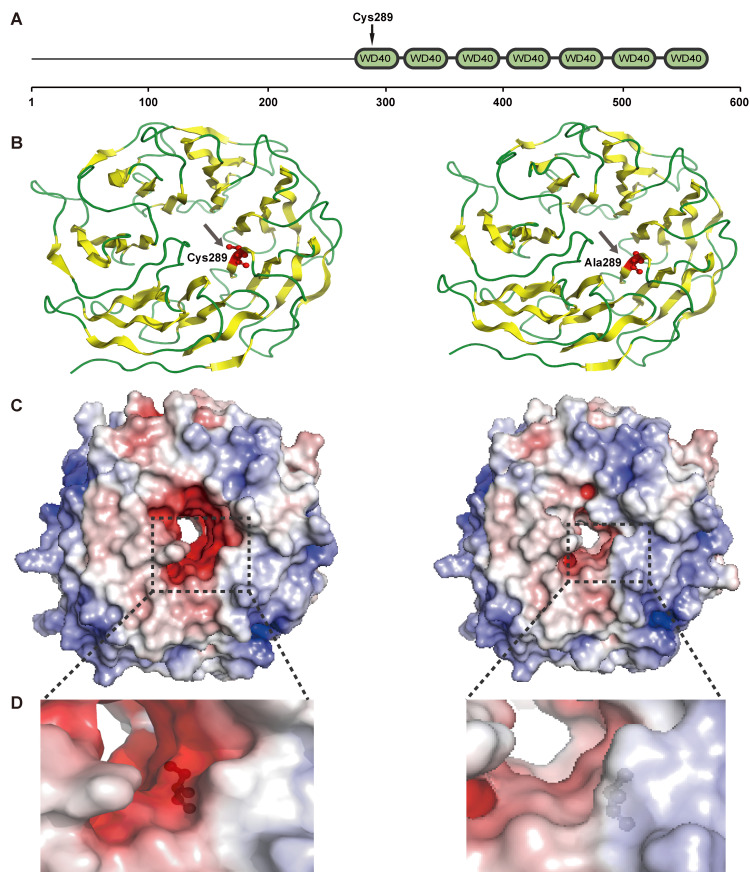
Structure of PRPF17 protein sequence of the *P. acdestis* (left) and *S. montelus* (right), respectively. (**A**): The PRPF17 protein domain with seven repeated WD40 motifs; the candidate positively selected site is indicated by black arrow. (**B**): The structures of sheet and loop are shown in yellow and green, respectively, with the positively selected residues shown with the red ball and stick representation. (**C**): Electrostatic potential map of PRPF17 domain; the range of electrostatic surface potential is shown from −7 kT/e (red color) to +7 kT/e (blue color). (**D**): The zoomed-in picture of electrostatic potential around the positively selected residues. Template in the Protein Data Bank: 5mqf.1.

Two of the candidate PSGs, ILF2 (interleukin enhancer-binding factor 2) and PRPF17 (pre-mRNA processing factor 17), are both involved in genetic information processing. ILF2 (also known as NF45) is a regulator of RNA splicing and DNA damage response that promotes mRNA processing and transcripts stabilization involved in homologous recombination [54], and dimerizes with ILF3 (also known as NF90) to form a protein complex involved in a variety of cellular processes [55]. The candidate positively selected amino acid mutation (G149Q) of ILF2 in swallowtail butterflies is located within the DZF domain (Supplementary Table S7 and Figure S6), likely altering its affinity to ILF3 and the protein complex activity in RNA splicing and DNA damage response. PRPF17 encodes a structurally and functionally conserved protein for pre-mRNA splicing and is related to the cell cycle progression [56], required for splicing-dependent NMD (nonsense mediated mRNA decay), serving to increase fidelity and efficiency of gene expression and to promote organism’s viability and development [57]. The A289C mutation of PRPF17 is found to be located at the first one of seven repeated WD40 motifs, which are known to serve as platforms for the assembly of protein complexes or mediators of transient interplay among other proteins [58]. Due to the mutation of the hydrophobic alanine to a hydrophilic cysteine, it could change the regional electrostatic potential as shown by comparison of the electrostatic potential maps (Figure 3), and is likely to form the disulfide bonds. It is probable that the A289C substitution is contributed to the protein-protein interaction involved in diverse functions.

The other two candidate PSGs, SHF (protein shifted or WIF-1-like protein) and CDASE (neutral ceramidase), are both involved in the signal transduction associated with environmental information processing. Serving as a component in the *Drosophila* Hedgehog signaling pathway, SHF is required for both stability and diffusion of cholesterol-modified Hedgehog, crucial to the morphogenesis and patterning (e.g., the wing development) [59]. All three positively selected sites of SHF here are located in the fourth epidermal growth factor (EGF)-like domain, different from the missense mutations between two known *Shf* alleles in the third EGF-like domain (see Figure 1B,C in Glise et al. [60]), implying that the positive selection of these selected sites and alleles resulted from different environmental conditions. CDASE plays important roles in insect development and stress responses (e.g., starvation, abnormal temperature, ultraviolet radiation and insecticides) through regulating the biosynthesis of sphingolipids [61], and in maintaining the photoreceptor homeostasis associated with UV damage response for *Drosophila* [62]. Two candidate positively selected mutations (the probable R307V and H605A) of CDASE are located in the N-terminal of the ZnF_CHCC domain and the highly conserved C-terminal tail of the enzyme, respectively (Supplementary Table S7 and Figure S7). According to the changes of hydrophobic properties and electrostatic pattern after ionization, these two mutations of CDASE may alter the affinity for zinc ion binding, and play important roles in stabilization and interaction of the enzyme with the plasma membranes [63].

The last one candidate PSG, PGM2 (phosphoglucomutase 2), plays an important role in the regulation of glycogen biosynthesis associated with the energy metabolism and stress resistance for environmental adaptation [64]. The positively selected site of PGM2 herein is located adjacent to the phosphorylation site of the domain 4 compared to the PGM1, and is probably associated with efficient catalysis [65]. Interestingly, the similar induced physicochemical change has been found in other proteins of the α-D-phosphohexomutase superfamily [66], and the positive selection of this gene was also detected in high-altitude dwelling *Phrynocephalus* and *Rana* lineages [2,4].

## 4. Discussion

The *Parnassius* internal phylogeny were not fully resolved, mainly due to the usage of only a limited number of molecular markers in previous studies, regardless of potentially homoplastic morphological characters used or not [12,13,14]. The main unresolved issues are as follows: (1) The equivocal position of its subgenus *Parnassius*, an important lineage for ancestral hostplant inference and biogeographic reconstruction; (2) unstable relationships of other subgenera due to the short branches on the trees, with similar mitochondrial and nuclear sequence datasets used in previous analyses [12,13,14,46]. Therefore, further efforts should focus on more inclusive sampling of genes as well as more *Parnassius* species [12]. However, previous studies indicated that the phylogenetic tree topology of *Parnassius* remained unresolved, even by sampling as many *Parnassius* species as possible (14,15,46). Rapid advances of RNA-Seq technologies have significantly accelerated the speed of gene gathering and genomics studies at reasonable cost. In this work, the *Parnassius* phylogenetic trees were reconstructed based on a large-scale transcriptomic datasets for the first time, using multiple analytic methods. Our results showed that all the topologies were basically the same with strong supports on nodes, regardless of the selection of sequences (nucleotides or amino acids), models, and tree-constructing methods (Figure 1, Supplementary Figure S5). These results indicated that high-throughput transcriptomic sequencing could represent a powerful approach to phylogenomics in a group that has previously been hardly to resolve.

Independent evidence indicated that the progressive extension of the QTP uplifts occurred during the Miocene period, responsible for the origin and radiation of the current biota in the region [48,49,67]. For the lineage of *Parnassius*, the significant up-shift in speciation rates has been detected, coincident with the colonization to the QTP [14], which can partially be explained by the QTP uplifts of providing novel, high-altitude habitats for the colonization of *Parnassius* common ancestor. Nevertheless, one of the premises of the QTP colonization for *Parnassius* species should be that their hostplants survived and diversified in the QTP region no later than its herbivores. This kind of temporal correlation between butterflies and their host plants has indirectly been corroborated by a survey, which indicated that butterflies along the Andean slope between 2400 and 5000 m acclimation to novel habitats depends on their larval hostplant use [68]. Previous phylogeographic studies indicated the *Corydalis* (Papaveraceae) colonized the QTP and Central Asia at about 37–26 Ma in Oligocene, the *Sedum* and *Rhodiola*-*Pseudosedum* clade (Crassulaceae) as well as *Saxifraga* (Saxifragaceae) originated in the QTP region at about 28.0–21.0 Ma [50,51,52]. Our dating estimates using transcriptomic data showed that the subgenus *Parnassius* (feeding on Crassulaceae+Saxifragaceae) diverged from other subgenera (feeding on Papaveraceae) probably in the Miocene (95% CI 7.7–24.0 Ma, Figure 1 and Table 1). Therefore, based on multiple independent studies above, the diversification pattern of *Parnassius* lineages are compatible with the spatiotemporal distribution pattern of their hostplants, supporting the underlying coevolutionary scenario between *Parnassius* and hostplants [69,70,71], mainly under the coupled effect of common extrinsic factors (e.g., mountain building, climate change and the resultant aridification event) in the Miocene [14]. Similar diversification patterns have also been found in alpine butterflies of the genus *Erebia* [72], the *arcesia* and *fergana* species groups in the genus *Melitaea* [6], and other animal groups [7,73]. Interestingly, the time range for diversification of *Parnassius* lineages is nearly contemporary with that of recent angiosperms’ radiation in western China (15.29–18.86 Ma) [5]. The coevolutionary scenario between *Parnassius* and hostplants can serve as a theoretical basis of conservation strategy for *Parnassius* and other alpine butterflies.

Multiple extrinsic factors can drive diversification of *Parnassius* lineages; however, currently little is known about the intrinsic genetic basis of the high-altitude adaptation for this butterfly genus. In this work, analyses of evolutionary rate estimation suggested that accelerated evolution occurred in the process of subgeneric origin and diversification in this alpine *Parnassius* butterfly group. Similar results of genome-wide rapid evolution were also found in Tibetan fishes, highland-dwelling lizard and ranid frog lineages [4,37,38]. In addition, two of the candidate PSGs here along the *Parnassius* lineages, PRPF17 and PGM2, seem to be homologous with those (PRPF4B, PGM2, PGM2L1, and PGM5, respectively) detected along the highland-dwelling lizard and frog lineages [2,4]. Therefore, both the accelerated evolution and the partial overlap of PSGs as well as related functions of all five PSGs herein, especially DNA damage response and energy metabolism pathways, probably exhibit numerous convergent evolutionary changes among different animal groups relating to high altitude adaptation. Nevertheless, three PSGs herein (ILF2, SHF, and CDASE), involved in insect development, morphogenesis, and stress responses (e.g., starvation, abnormal temperature, ultraviolet radiation, and DNA damage), are not shared with previously reported genes in other animals. This observation suggests that *Parnassius* butterflies may have employed different genetic toolkits to adapt to extreme and substantially variable environments of the QTP, and can serve as a foundation for future studies aiming to mitigate the threats to alpine butterflies posed by climate changes and environmental disruptions. However, this hypothesis needs to be further confirmed by population genomics studies in the future.

Overall, at least several intrinsic traits may contribute to the *Parnassius* adaptive evolution: The relatively faster evolutionary rates for subgeneric origin than those of other low-altitude papilionids revealed by genome-wide elevated dN/dS ratios; the five detected PSGs with functions as mentioned above in promoting stabilization of genetic information processing for viability and development, stress responses (e.g., DNA damage, starvation, abnormal temperature, and ultraviolet radiation), and energy metabolism, et al., suggesting their multifaceted strategies for long-lasting adaptation to high-altitude environments of low level of oxygen, low temperature and strong ultraviolet radiation. In the future, samplings of additional taxa and more high-quality genomic data are needed to clarify the evolutionary spatiotemporal pattern of *Parnassius* butterflies and the underlying extrinsic and intrinsic factors more effectively.

## 5. Conclusions

Our large-scale transcriptomic data of eight representative *Parnassius* species and their two closely related papilionid species are valuable in establishing well-resolved phylogeny and a more reasonable timescale for the divergences of genus *Parnassius*, which initiated in the middle Miocene, followed by successive subgeneric diversifications that may be historically correlated with their hostplant spatiotemporal distributions as well as the geological and environmental changes in the QTP region. Additionally, our study provides the first report about the genetic mechanism of QTP high-altitude adaptation of the main *Parnassius* butterfly lineages at the whole genome scale, that is, the accelerated evolution for subgeneric origin and diversification correlated with hostplant shifts, candidate PSGs and their potentially functional changes may attribute to both the convergent evolution and the unique strategies for long-lasting adaptation to high-altitude environments, which will help interested researchers to address evolution, biodiversity and conservation questions concerning *Parnassius* and other butterfly species.

## Figures and Tables

**Table 1:** Comparison of crown ages with the 95% confidence interval (CI) of major lineages and the most recent common ancestor (MRCA) of major splits in genus *Parnassius* across different datasets.

Major Lineage/Split	Amino Acid Dataset		Nucleotide Dataset	
	Mean Age	95% CI	Mean Age	95% CI
Genus *Parnassius*	14.3	7.7–24.0	13.0	6.8–22.3
*Tadumia* + *Kailasius*/*Kreizbergia* + *Driopa*	12.1	6.4–20.4	11.2	5.9–19.3
*Tadumia*/*Kailasius*	10.8	5.8–18.6	9.9	5.2–17.1
*Kreizbergia*/*Driopa*	11.1	6.0–18.8	10.2	5.3–17.5

**Table 2:** Results of positive selection analysis for the branch of the genus *Parnassius.*

Gene	Model with Positive Selection (Model A)			Null Model (Model A null)		LRT	*p*-Value	Biological Category
	L_1_	Parameters	Positively Selected Sites	L_0_	Parameters	2ΔL		
*ILF2*	−1335.05	k = 1.73	Ser149Gln 0.98 *	−1337.71	k = 1.71	5.32	0.02	RNA splicing and DNA damage response
		p_0_ = 0.97			p_0_ = 0.95			
		ω_0_ = 0.02			ω_0_ = 0.02			
		p_s_ = 0.02						
		ω_s_ = 25.86						
*PRPF17*	−4662.37	k = 1.65	Ala289Cys 0.98 *	−4664.23	k = 1.64	3.72	0.05	pre-mRNA splicing and mRNA surveillance
		p_0_ = 0.97			p_0_ = 0.95			
		ω_0_ = 0.03			ω_0_ = 0.03			
		p_s_ = 0.01						
		ω_s_ = 14.87						
*SHF*	−3105.63	k = 2.04	Val300Arg 1.00 **	−3108.38	k = 2.02	5.50	0.02	morphogenesis and patterning
		p_0_ = 0.94	Lys304Arg 0.98 *		p_0_ = 0.91			
		ω_0_ = 0.02	Asp314Arg 0.98 *		ω_0_ = 0.02			
		p_s_ = 0.03						
		ω_s_ = 5.34						
*CDASE*	−11227.05	k = 1.95	Arg307Val 0.97 *	−11229.24	k = 1.94	4.38	0.03	development and stress responses
		p_0_ = 0.92	His605Ala 0.98 *		p_0_ = 0.91			
		ω_0_ = 0.05			ω_0_ = 0.05			
		p_s_ = 0.01						
		ω_s_ = 9.96						
*PGM2*	−5402.44	k = 1.79	Cys504Thr 1.00 **	−5406.12	k = 1.77	7.36	0.01	glycogen metabolism and environmental adaptation
		p_0_ = 0.88			p_0_ = 0.86			
		ω_0_ = 0.06			ω_0_ = 0.06			
		p_s_ = 0.01						
		ω_s_ = 15.05						

* if the posterior probability is >0.95, and ** if the probability is >0.99 in the Bayes empirical Bayes (BEB) calculation; the numerical order of positively selected sites referred to the corresponding genes of Bombyx mori; L_1_, Log-likelihood value in Model A; L_0_, Log-likelihood value in Model A null; LRT, likelihood ratio test; 2△L, equal to 2 × (L1-L0).

## Supplementary Materials

The following are available online at https://www.mdpi.com/2075-4450/11/11/754/s1, Figure S1: Results of annotation in the NR database for *Parnassius* species, Figure S2: Characteristics of GO term of biological process, cellular component and molecular function for *Parnassius* species, Figure S3: Characteristics of eggNOG functional category and KEGG pathway classification of unigenes for *Parnassius* species, Figure S4: BUSCO analysis of transcriptome completeness, Figure S5: The species-level *Parnassius* phylogenetic relationships using maximum likelihood and Bayesian inference methods, Figure S6: Structure of ILF2 protein sequence of the *P. acdestis* (left) and *S. montelus* (right), respectively, Figure S7: Structure of CDASE protein sequence of the *P. acdestis* (left) and *S. montelus* (right), respectively, Table S1: Information of samples used for transcriptome sequencing, Table S2: Substitution model of each partition with different partitioning schemes, Table S3: Summary of assembly results of transcriptome data for species, Table S4: Summary of functional annotation of Unigenes, Table S5: Results of cross-contamination check using the CroCo program, Table S6: Overview of the positively selected genes and their quality assessment, Table S7: Amino acid substitution specificity information for positively selected sites along the lineage leading to the genus *Parnassius*, Dataset S1: Phylogenomic dataset including 476 orthologous genes in nucleotides format.
