# Supplementary material for: Miocene Diversification and High-Altitude Adaptation of Parnassius Butterflies (Lepidoptera: Papilionidae) in Qinghai–Tibet Plateau Revealed by Large-Scale Transcriptomic Data
Source: Insects. 2020 Nov 3;11(11):754. doi: 10.3390/insects11110754 (PMC7693471; doi:10.3390/insects11110754)
Supplement: Supplementary file 1 [file insects-11-00754-s001.zip › insects-956853-Supplementary-figures.pdf]

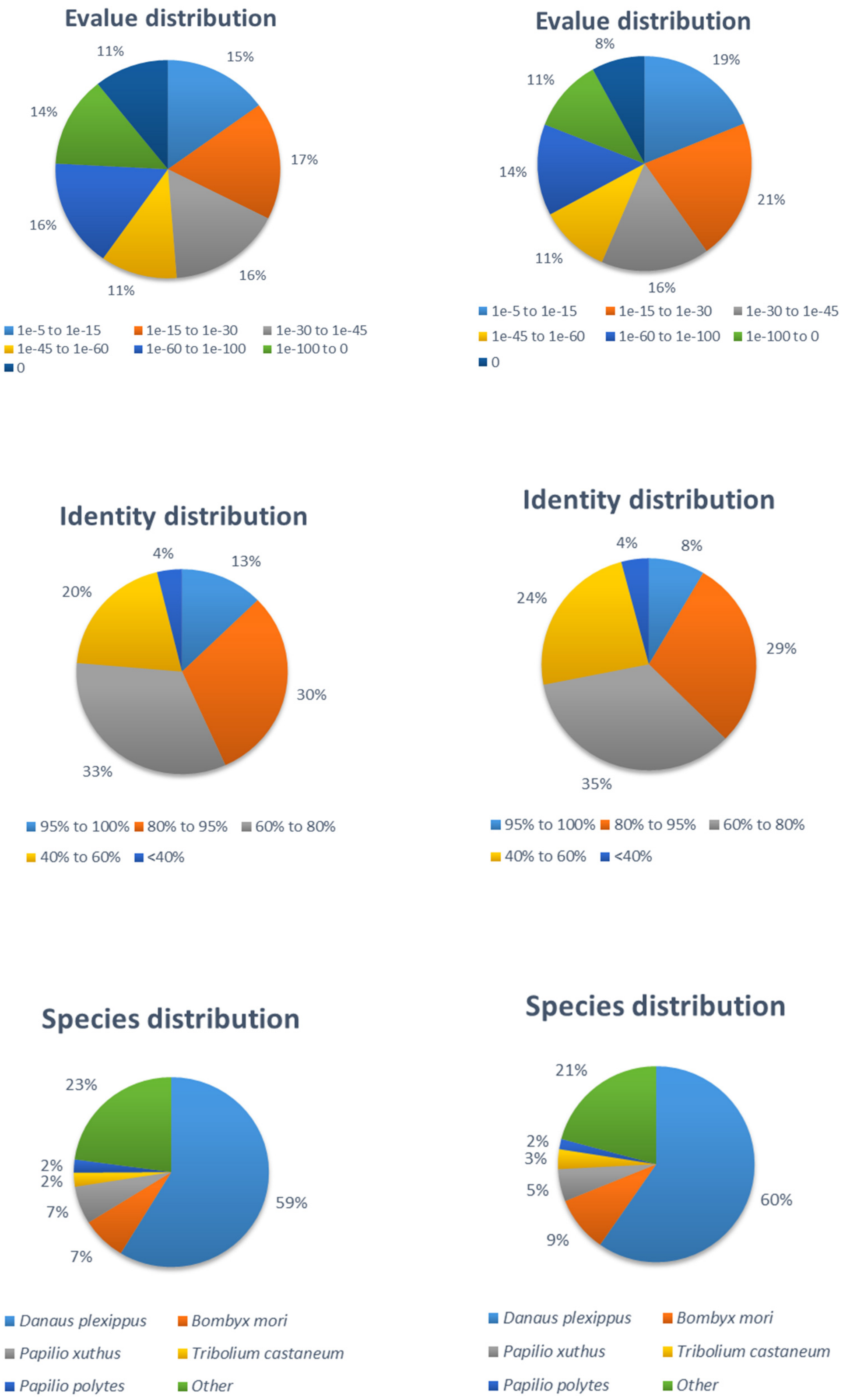

Figure 1. Results of annotation in the NR database for *Parnassius* species (*P. glacialis* (left) and *P. epaphus* (right) as examples).

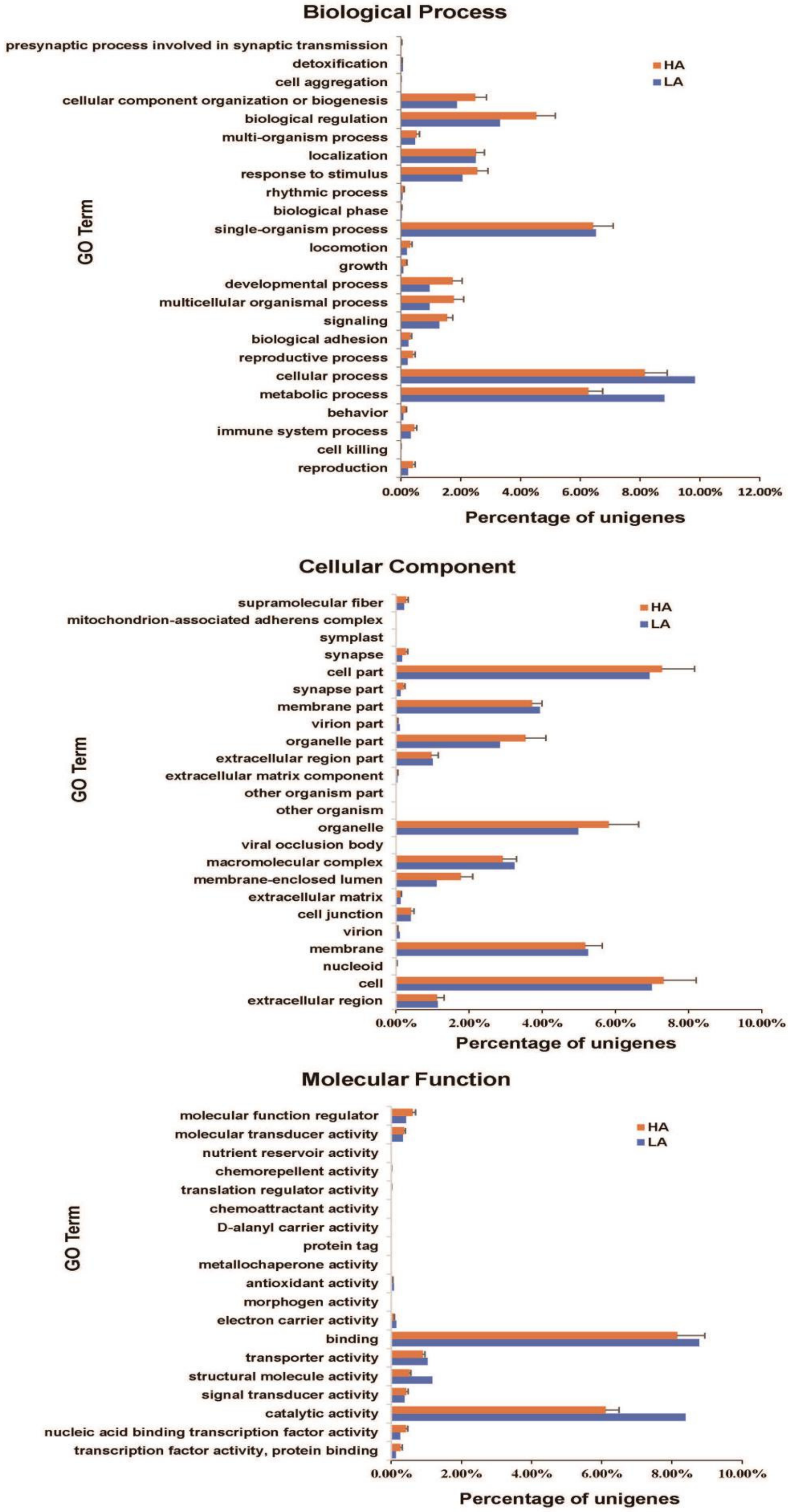

Figure 2. Characteristics of GO term of biological process, cellular component and molecular function for *Parnassius* species (HA, *Parnassius* species colonized at high-altitude habitats except *Parnassius glacialis*; LA, *Parnassius glacialis* colonized at low-altitude habitats).

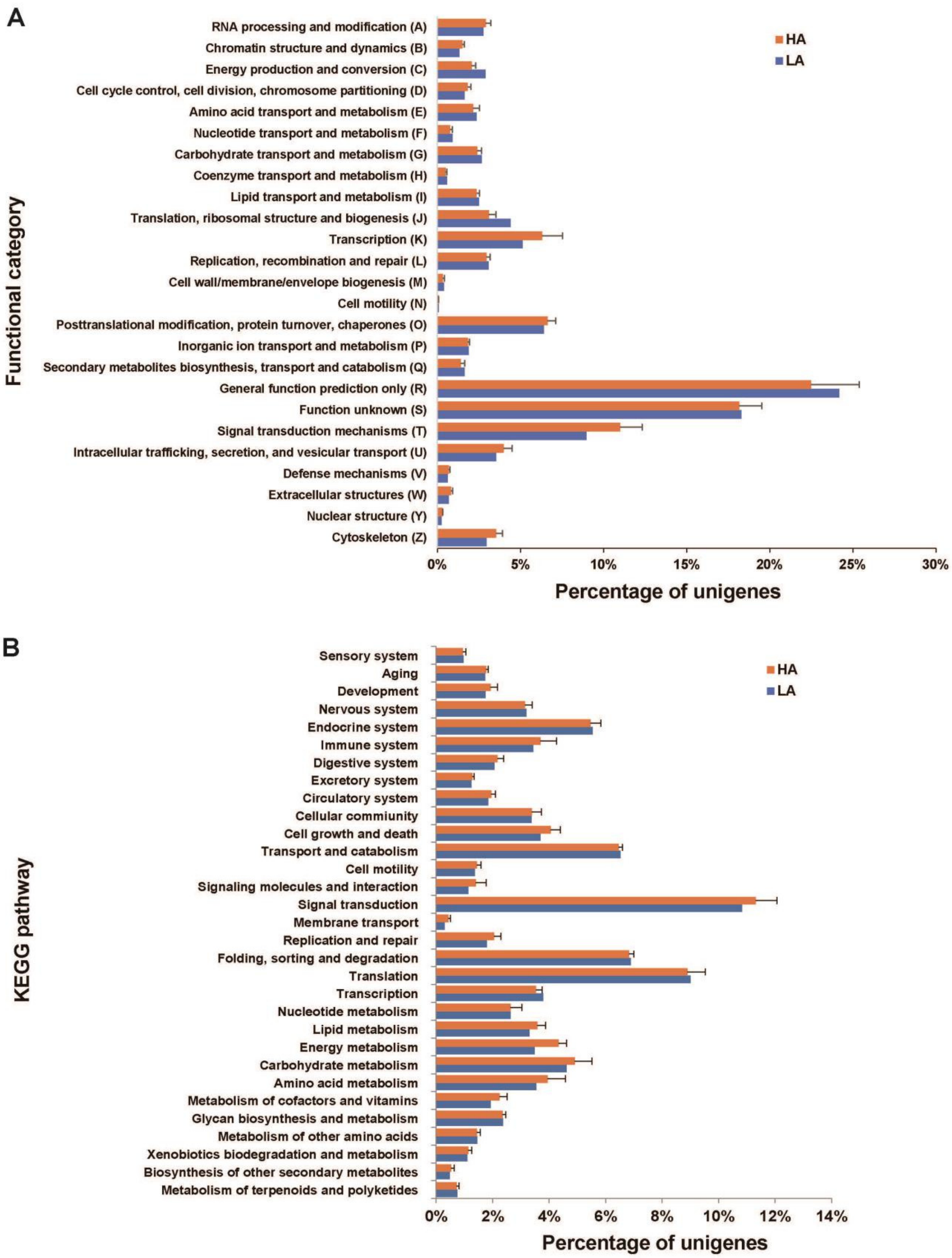

Figure 3. Characteristics of eggNOG functional category (A) and KEGG pathway classification (B) of unigenes for *Parnassius* species.

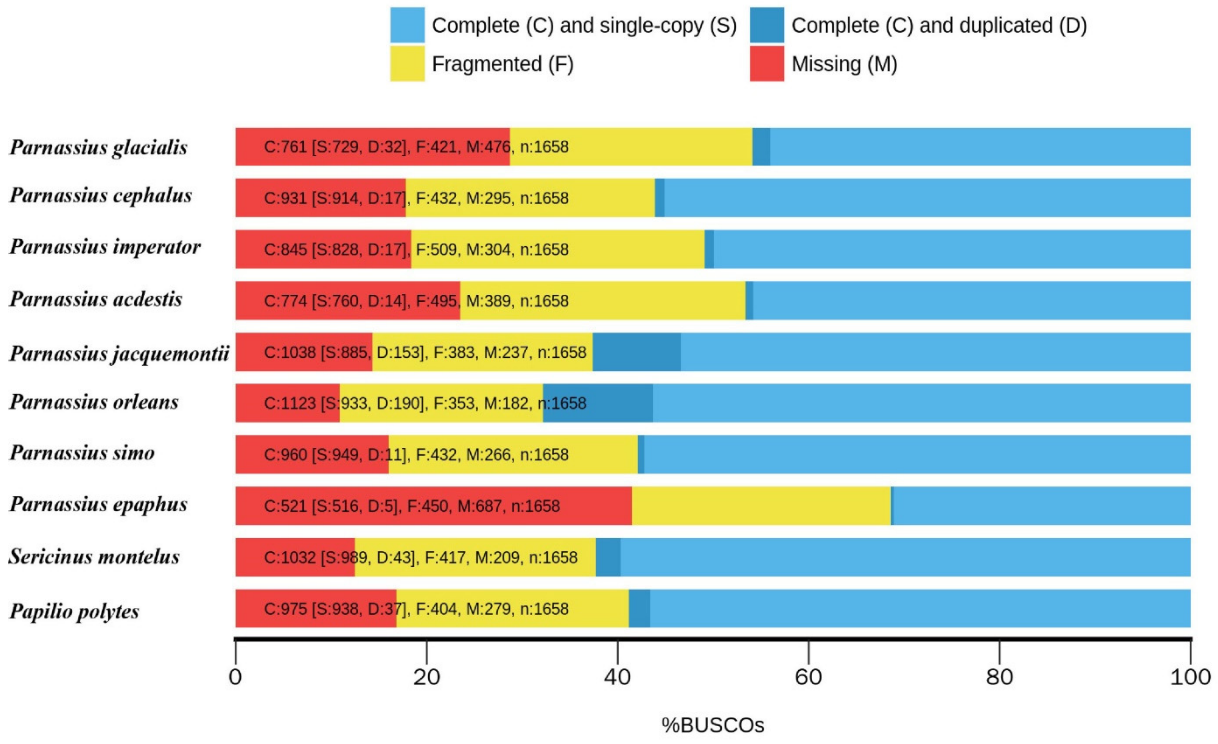

Figure 4. BUSCO analysis of transcriptome completeness. BUSCO v4.0.2 was run using insecta_odb9 dataset.

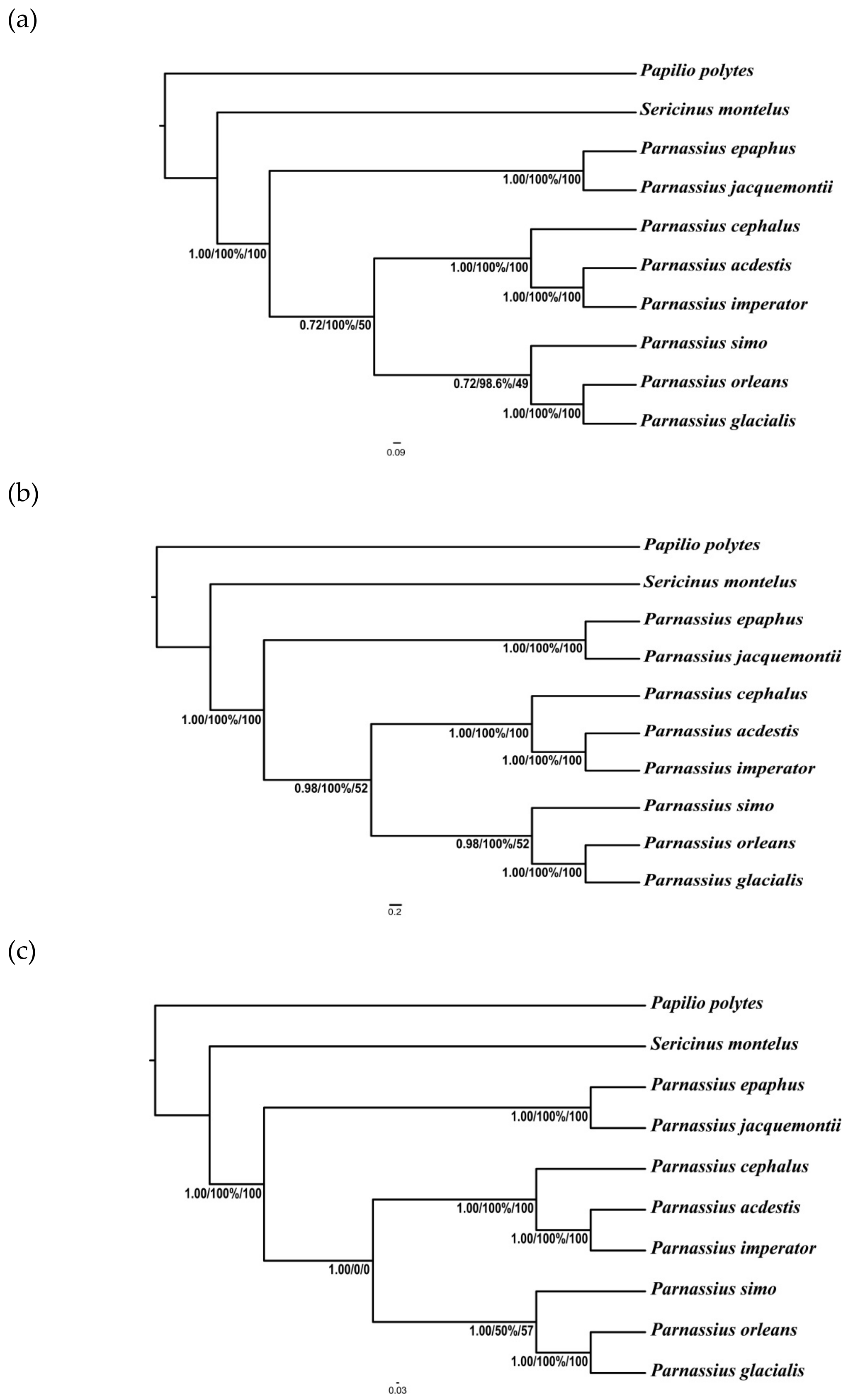

Figure 5. The species-level *Parnassius* phylogenetic relationships using maximum likelihood and Bayesian inference methods, (a) based on amino acid sequence and the best-fit amino acid model; (b) based on nucleotide sequence and the best-fit codon model; (c) based on nucleotide sequence and the best-fit nucleotide model. The PP (posterior probabilities), SH-aLRT and UFBoot support values are shown sequentially for each node.

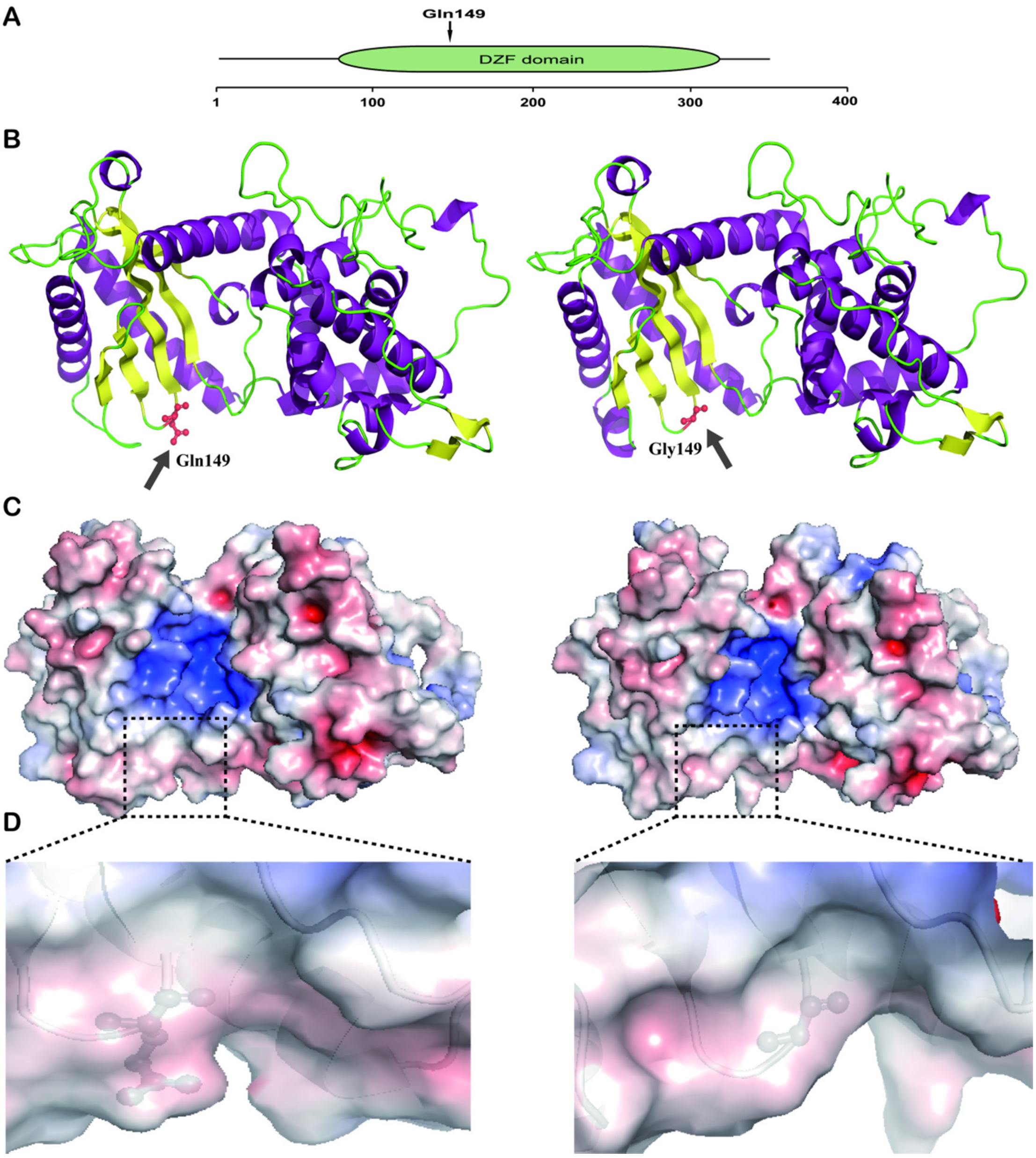

Figure 6. Structure of ILF2 protein sequence of the *P. acdestis* (left) and *S. montelus* (right), respectively. A: The ILF2 protein domain with DZF domain; the candidate positively selected site is indicated by black arrow. B: the structures of helix, sheet and loop are shown in purple, yellow and green, respectively, with the positively selected residues shown with the red ball and stick representation. C: Electrostatic potential map of ILF2 domain; the range of electrostatic surface potential is shown from −7 kT/e (red color) to +7 kT/e (blue color). D: the zoomed-in picture of electrostatic potential around the positively selected residues. Template in the Protein Data Bank: 4atb.1.

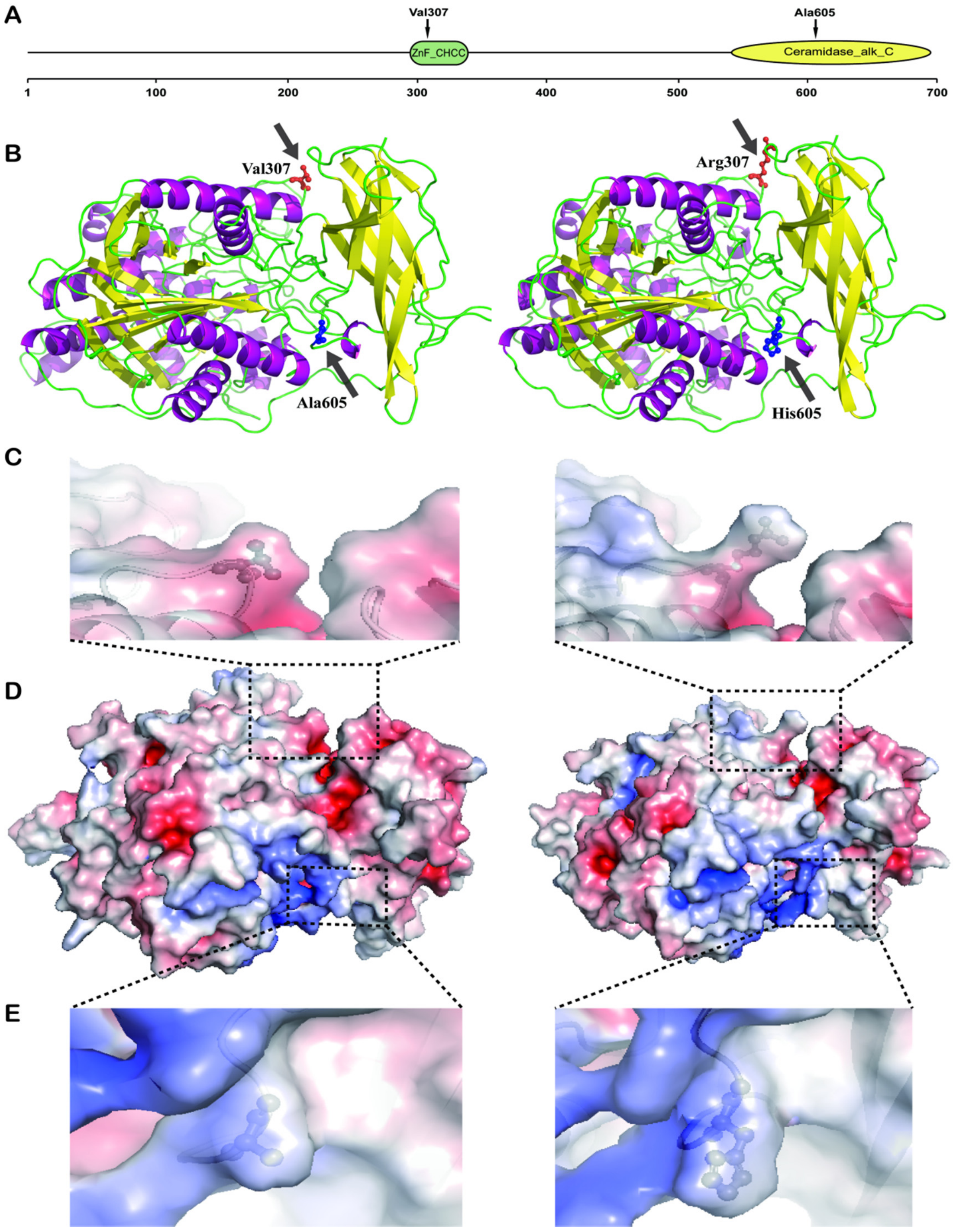

Figure 7. Structure of CDASE protein sequence of the *P. acdestis* (left) and *S. montelus* (right), respectively. A: The CDASE protein domain with the ZnF_CHCC domain and the highly conserved C-terminal tail (ceramidase_alk_C); two candidate positively selected sites are indicated by black arrows. B: the structures of helix, sheet and loop are shown in purple, yellow and green, respectively, with the positively selected residues shown with the red ball and stick representation. D: Electrostatic potential map of CDASE domain. C and E: the zoomed-in pictures of electrostatic potential around the positively selected residues. Template in the Protein Data Bank: 4wgk.1.
