# Supplementary material for: Miocene Diversification and High-Altitude Adaptation of Parnassius Butterflies (Lepidoptera: Papilionidae) in Qinghai–Tibet Plateau Revealed by Large-Scale Transcriptomic Data
Source: Insects. 2020 Nov 3;11(11):754. doi: 10.3390/insects11110754 (PMC7693471; doi:10.3390/insects11110754)
Supplement: Supplementary file 1 [file insects-11-00754-s001.zip › insects-956853-Supplementary-tables.pdf]

Table 1. Information of samples used for transcriptome sequencing.

NO.	Subfamily/Tribe /Genus	Subgenus	Species	Main Host plant	Sampling Location	Latitude/ Longitude	Main Altitude Distribution
1	Parnassiinae/Parnassiini /*Parnassius*	*Parnassius*	*Parnassius epaphus*	Crassulaceae (*Rhodiola, Sedum*)	Gansu, China	N39.013/ E98.888	3,500 m～5,500 m, alpine
2	Parnassiinae/Parnassiini /*Parnassius*	*Parnassius*	*Parnassius jacquemontii*	Crassulaceae (*Rhodiola*)	Qinghai, China	N36.465/ E102.087	3,500 m～5,000 m, alpine
3	Parnassiinae/Parnassiini /*Parnassius*	*Tadumia*	*Parnassius cephalus*	Papaveraceae (*Corydalis*)	Qinghai, China	N38.089/ E100.658	>3,500 m, alpine
4	Parnassiinae/Parnassiini /*Parnassius*	*Kailasius*	*Parnassius imperator*	Papaveraceae (*Corydalis*)	Gansu, China	N34.302/ E103.207	2,800 m～3500 m, alpine
5	Parnassiinae/Parnassiini /*Parnassius*	*Kailasius*	*Parnassius acdestis*	Papaveraceae (*Corydalis*)	Gansu, China	N34.269/ E103.207	4,000 m～4,500 m, alpine
6	Parnassiinae/Parnassiini /*Parnassius*	*Kreizbergia*	*Parnassius simo*	Scrophulariaceae and Papaveraceae (*Lagotis, Corydalis*)	Qinghai, China	N38.089/ E100.658	>4,000 m, alpine
7	Parnassiinae/Parnassiini /*Parnassius*	*Driopa*	*Parnassius orleans*	Papaveraceae (*Corydalis*)	Qinghai, China	N36.4653/E102.08704	2,500 m～4,000 m, alpine
8	Parnassiinae/Parnassiini /*Parnassius*	*Driopa*	*Parnassius glacialis*	Papaveraceae (*Corydalis*)	Jiangsu, China	N32.072/E118.837	200 m～1000 m, subalpine
9	Parnassiinae/Zerynthiini/*Sericinus*	-	*Sericinus montelus*	Aristolochiaceae	Anhui, China	N31.348/ E118.383	lowland
10	Papilioninae/Papilionini	-	*Papilio polytes*	Rutaceae	Anhui, China	N31.338/ E118.382	lowland

Table 2. Substitution model of each partition with different partitioning schemes.

Dataset	Scheme	Total Lenth	Substitution Model (AICc)	
			**ML**	**BI**
**Amino acid**	**unpartitioned**	145,077 aa, 476 genes	JTT+I+G	mixed
Codon	unpartitioned	145,077 codons, 476 genes	GY+F+R4	codon
Nucleotide	partitioned by codon positions	1st, 145,077 bp, 476 genes	GTR+I+G	GTR+I+G
		2nd, 145,077 bp, 476 genes	GTR+I	GTR+I
		3rd, 145,077 bp, 476 genes	GTR+G	GTR+G

Table 3. Summary of assembly results of transcriptome data for species.

Item	*Parnassius*								*Sericinus*	*papilio*	**Average of**
	P. glacialis	*P. orleans*	*P. imperator*	*P. acdestis*	*P. cephalus*	*P. simo*	*P. epaphus*	*P. jacquemontii*	*S. montelus*	*p. polytes*	***Parnassius***
Total number of clean reads	58,078,158	67,887,602	64,578,298	66,997,138	61,256,404	68,702,470	62,749,958	65,243,576	56,253,056	60,880,890	**64,436,701**
Total length of clean data (Gbp)	8.50	9.87	9.32	9.75	8.98	9.83	9.02	9.48	8.17	8.89	**9.34**
**Final Assembly**											
Total length of unigenes (Mbp)	18.20	32.34	16.92	16.55	23.20	22.91	17.85	30.14	29.36	19.10	**22.26**
Total number of unigenes	35,107	64,863	34,570	34,096	44,276	44,481	41,777	61,868	60,642	30,870	**45,130**
Maximum length of unigenes (bp)	18,973	14,591	7,711	10,693	10,026	9,118	11,167	15,257	13,786	13,414	**12,192**
Mean length of unigenes (bp)	518	499	489	485	524	515	427	487	484	618	**493**
N50 length of unigenes (bp)	658	609	585	584	655	648	452	584	551	925	**597**
Number of N50 sequences	6,747	13,903	7,775	7,443	9,546	9,302	9,302	13,193	12,765	5,441	**9,651**
GC%	47.39	48.25	44.97	45.07	44.84	44.36	47.63	49.08	47.94	48.98	**46.45**
Total number of predicted proteins	19,371	39,521	20,105	18,585	24,288	24,201	19,165	37,545	30,627	19,845	**25,348**

bp = base pair; Gbp = billion base pairs; Mbp = mega base pairs.

Table 4. Summary of functional annotation of Unigenes.

Database	*Parnassius*																*Sericinus*		*papilio*		**Average(*Parnassius*)**	
	P. glacialis		*P. orleans*		*P. imperator*		*P. acdestis*		*P. cephalus*		*P. simo*		*P. epaphus*		*P. jacquemontii*		*S. montelus*		*p. polytes*			
**Annotated**	Number	Percent(%)	Number	Percent(%)	Number	Percent(%)	Number	Percent(%)	Number	Percent(%)	Number	Percent(%)	Number	Percent(%)	Number	Percent(%)	Number	Percent(%)	Number	Percent(%)	**Number**	**Percent(%)**
NR	18,923	53.90	39,121	60.31	19,993	57.83	18,383	53.92	22,506	50.83	23,163	52.07	19,059	45.62	37,329	60.34	27,850	45.93	19,434	62.95	**24,810**	**54.35**
Swissprot	12,505	35.62	30,817	47.51	12,366	35.77	11,687	34.28	16,784	37.91	15,670	35.23	10,728	25.68	29,546	47.76	23,468	38.70	13,659	44.25	**17,513**	**37.47**
KEGG	4,415	12.58	10,086	15.55	5,243	15.17	4,948	14.51	6,545	14.78	6,158	13.84	4,326	10.35	9,563	15.46	7,104	11.71	5,572	18.05	**6,411**	**14.03**
eggNOG	19,018	54.17	38,510	59.37	20,312	58.76	18,434	54.06	22,697	51.26	23,529	52.90	19,076	45.66	36,509	59.01	27,865	45.95	19,498	63.16	**24,761**	**54.40**
GO	11,876	33.83	29,468	45.43	12,349	35.72	11,497	33.72	13,456	30.39	13,816	31.06	11,158	26.71	28,820	46.58	18,441	30.41	12,760	41.33	**16,555**	**35.43**
In all database	3,605	10.27	9,025	13.91	4,201	12.15	3,998	11.73	4,733	10.69	4,636	10.42	3,478	8.33	8,698	14.06	5,452	8.99	4,343	14.07	**5,297**	**11.45**
Total annotated	19,887	56.65	40,208	61.99	20,692	59.86	19,038	55.84	24,816	56.05	24,831	55.82	19,777	47.34	38,011	61.44	31,237	51.51	20,252	65.60	**25,908**	**56.87**
**Unannotated**	15,220	43.35	24,655	38.01	13,878	40.14	15,058	44.16	19,460	43.95	19,650	44.18	22,000	52.66	23,857	38.56	29,405	48.49	10,618	34.40	**19,222**	**43.13**
**Total**	35,107	100	64,863	100	34,570	100	34,096	100	44,276	100	44,481	100	41,777	100	61,868	100	60,642	100	30,870	100	**45,130**	**100**

Table 5. Results of cross-contamination check using the CroCo program.

species	total_unigenes	nb_suspects†	nb_clean†	nb_lowcov†	nb_overexp†	nb_dubious†	nb_contam†	clean_percent†	lowcov_percent	overexp_percent	dubious_percent	contam_percent
Parnassius glacialis	35107	15933	15512	63	8	266	84	98.8	0.17	0.02	0.75	0.23
Parnassius orleans	64863	37754	13364	16212	12	7251	915	62.39	24.99	0.01	11.17	1.41
Parnassius imperator	34570	16792	11439	2857	13	1033	1450	84.51	8.26	0.03	2.98	4.19
Parnassius simo	44481	16306	12601	2223	16	848	618	91.67	4.99	0.03	1.90	1.38
Parnassius acdestis	34096	13558	11482	1145	0	719	212	93.91	3.35	0	2.10	0.62
Parnassius cephalus	44276	13316	11559	907	0	654	196	96.03	2.04	0	1.47	0.44
Parnassius jacquemontii	61868	9155	7427	756	9	614	349	97.2	1.22	0.01	0.99	0.56
Parnassius epaphus	41777	9835	7009	1778	0	630	418	93.23	4.25	0	1.50	1.00
Sericinus montelus	60642	1780	1248	131	6	324	71	99.12	0.21	0	0.53	0.11
Papilio polytes	30870	2017	1040	109	8	605	255	96.83	0.35	0.02	1.95	0.82

†: nb_suspects, number of suspect unigenes; nb_clean, number of unigenes from the focal sample; nb_lowcov, number of unigenes with low expression levels; nb_overexp, number of unigenes with high expression levels; nb_dubious, number of unigenes with close expression levels between focal and alien samples; nb_contam, number of unigenes from an alien sample; clean_percent, percentage of unigenes from the focal sample; others similar.

Table 6. Overview of the positively selected genes and their quality assessment.

**Positively selected genes**	**Transcripts Per Million (TPM) †**		**Length of Alignment with no Indels**	**Coverage ‡**
	**Average**	**STDEV**		
***SHF* (protein shifted)**	**5.56**	5.00	966	85.64% (966/1128)
ILF2 (interleukin enhancer-binding factor 2)	14.90	8.54	1052	89.68% (1052/1173)
CDASE (neutral ceramidase)	8.94	7.35	2046	98.27% (2046/2082)
PGM2 (phosphoglucomutase 2)	11.39	10.03	1803	98.85% (1803/1824)
PRPF17 (pre-mRNA processing factor 17)	23.50	15.85	1365	79.13% (1365/1725)

†: Indicates the expression level in each focal sample using the CroCo program. If a transcript has lower TPM value than the threshold (default value: 0.2) in all samples it will then be categorized as a "low coverage" transcript. ‡: Relative coverage for each coding DNA sequence (CDS) of each species compared with CDS of orthologous gene of *Papilio xuthus* and *Papilio machaon*.

Table 7. Amino acid substitution specificity information for positively selected sites along the lineage leading to the genus *Parnassius*.

ILF2	Amino Acid Position 149 in the CDS of AQY61716																			
P. acdestis	**E**	**R**	**G**	**F**	**T**	**V**	**T**	**A**	**P**	**Q**	**A**	**A**	**V**	**R**	**V**	**L**	**V**	**T**	T	L
P. glacialis	E	R	G	F	T	V	T	A	P	Q	A	A	V	R	V	L	V	T	T	L
P. cephalus	E	R	G	F	T	V	T	A	P	Q	A	A	V	R	V	L	V	T	T	L
P. epaphus	E	R	G	F	T	V	T	A	P	Q	A	A	V	S	V	L	V	T	T	L
P. imperator	E	R	G	F	T	V	T	A	P	Q	A	A	V	R	V	L	V	T	T	L
P. jacquemontii	E	R	G	F	T	V	T	A	P	Q	A	A	V	S	V	L	V	T	T	L
P. orleans	E	R	G	F	T	V	T	A	P	Q	A	A	V	R	V	L	V	T	T	L
P. simo	E	R	G	F	T	V	T	A	P	Q	A	A	V	R	V	L	V	T	T	L
Sericinus montelus	E	R	G	F	T	V	S	A	P	G	A	A	V	R	V	L	V	T	T	L
papilio polytes	E	S	G	F	T	V	T	A	A	G	A	A	V	R	V	L	V	T	T	L
Papilio xuthus	E	R	G	F	T	V	T	A	A	G	A	A	V	R	V	L	V	T	T	L
Papilio machaon	E	R	G	F	T	V	T	A	A	G	A	A	V	R	V	L	V	T	T	L
Pieris rapae	E	R	G	F	T	V	T	A	A	S	S	A	V	R	V	L	V	T	T	L
Danaus plexippus	E	S	G	F	T	V	S	T	G	A	A	G	V	S	G	L	V	T	T	L
Bombyx mori	E	R	G	F	T	V	T	G	T	S	A	A	V	S	V	L	I	T	T	L
Spodoptera litura	E	R	G	F	T	V	T	G	P	S	A	A	V	S	V	L	V	T	T	L
Manduca sexta	E	R	G	C	T	V	T	G	A	A	A	A	V	R	L	L	V	T	T	L

PRPF17	Amino acid position 289 in the CDS of XP_004922916																			
P. acdestis	T	W	K	G	H	S	K	G	V	A	C	V	R	W	Y	P	S	T	A	H
P. glacialis	T	W	K	G	H	T	K	G	V	A	C	V	R	W	F	P	S	T	A	H
P. cephalus	T	W	K	G	H	S	K	G	V	A	C	V	R	W	F	P	S	T	A	H
P. epaphus	T	W	K	G	H	S	K	G	V	A	C	V	R	W	F	P	S	T	A	H
P. imperator	T	W	K	G	H	S	K	G	V	A	C	V	R	W	Y	P	S	T	A	H
P. jacquemontii	T	W	K	G	H	S	K	G	V	A	C	V	R	W	F	P	S	T	A	H
P. orleans	T	W	K	G	H	T	K	G	V	A	C	V	R	W	F	P	S	T	A	H
P. simo	T	W	K	G	H	S	K	G	V	A	C	V	R	W	F	P	S	T	A	H
Sericinus montelus	T	W	K	G	H	T	K	G	V	A	A	V	R	W	F	P	R	T	A	H
papilio polytes	T	W	R	G	H	T	K	G	V	A	A	A	R	W	L	P	A	T	A	H
Papilio xuthus	T	W	K	G	H	T	K	G	V	A	A	V	R	W	L	P	S	T	A	H

Papilio machaon	T	W	K	G	H	T	K	G	V	A	A	V	R	W	L	P	S	T	A	H
Pieris rapae	T	W	K	G	H	T	K	G	V	A	A	V	R	W	F	P	N	T	A	H
Danaus plexippus	T	W	K	G	H	T	K	G	V	S	A	V	R	W	F	P	R	T	A	H
Bombyx mori	T	W	K	G	H	T	K	G	V	A	A	A	R	W	F	P	S	T	A	H
Spodoptera litura	T	W	K	G	H	T	K	G	V	A	A	V	R	W	F	P	G	T	A	H
Manduca sexta	T	W	R	G	H	T	K	G	V	A	A	A	R	W	F	P	R	T	A	H

SHF	Amino acid position 300, 304, 314 in the CDS of ABD36274																			
P. acdestis	R	C	R	G	V	N	R	C	R	C	P	A	G	L	G	G	R	H	C	E
P. glacialis	R	C	R	G	V	N	R	C	R	C	P	A	G	L	G	G	R	H	C	E
P. cephalus	R	C	R	G	V	N	R	C	R	C	P	A	G	L	G	G	R	H	C	E
P. epaphus	R	C	R	G	V	N	R	C	R	C	P	A	G	L	G	G	R	H	C	E
P. imperator	R	C	R	G	V	N	R	C	R	C	P	A	G	L	G	G	R	H	C	E
P. jacquemontii	R	C	R	G	V	N	R	C	R	C	P	A	G	L	G	G	R	H	C	E
P. orleans	R	C	R	G	V	N	R	C	R	C	P	A	G	L	G	G	R	H	C	E
P. simo	R	C	R	G	V	N	R	C	R	C	P	A	G	L	G	G	R	H	C	E
Sericinus montelus	R	C	K	G	V	N	K	C	R	C	P	P	G	L	G	G	N	H	C	E
papilio polytes	R	C	K	G	V	N	K	C	R	C	P	P	G	L	G	G	D	H	C	E
Papilio xuthus	R	C	K	G	V	N	K	C	R	C	P	P	G	L	G	G	D	H	C	E
Papilio machaon	R	C	K	G	V	N	K	C	R	C	P	P	G	L	G	G	D	H	C	E
Pieris rapae	R	C	K	G	V	N	K	C	R	C	P	P	G	L	G	G	N	H	C	E
Danaus plexippus	R	C	K	G	V	N	K	C	R	C	P	A	G	L	G	G	N	H	C	E
Bombyx mori	R	C	V	G	V	N	K	C	R	C	P	A	G	L	G	G	D	H	C	E
Spodoptera litura	R	C	K	G	V	N	K	C	R	C	P	V	G	L	G	G	N	H	C	E
Manduca sexta	R	C	K	G	V	N	K	C	R	C	P	A	G	L	G	G	N	H	C	E

CDASE	Amino acid position 307 in the CDS of XP_004929418																			
P. acdestis	K	E	R	C	F	A	S	G	P	G	V	D	M	F	D	S	T	R	I	I
P. glacialis	K	E	R	C	F	A	S	G	P	G	V	D	M	F	D	S	T	R	I	I
P. cephalus	K	E	R	C	F	A	S	G	P	G	V	D	M	F	D	S	T	R	I	I
P. epaphus	K	E	R	C	F	A	S	G	P	G	V	D	M	F	D	S	T	R	I	I
P. imperator	K	E	R	C	F	A	S	G	P	G	V	D	M	F	D	S	T	R	I	I

P. jacquemontii	K	E	R	C	F	A	S	G	P	G	V	D	M	F	D	S	T	R	I	I
P. orleans	K	E	R	C	F	A	S	G	P	G	V	D	M	F	D	S	T	R	I	I
P. simo	K	E	R	C	F	A	S	G	P	G	V	D	M	F	D	S	T	R	I	I
Sericinus montelus	K	E	R	C	F	A	A	G	P	G	R	D	M	F	D	S	T	R	I	I
papilio polytes	K	E	R	C	F	A	S	G	P	G	S	D	M	F	D	S	T	R	I	I
Papilio xuthus	K	E	R	C	F	A	S	G	P	G	R	D	M	F	D	S	T	R	I	I
Papilio machaon	K	E	R	C	F	A	S	G	P	G	R	D	M	F	D	S	T	R	I	I
Pieris rapae	K	E	S	C	F	A	S	G	P	G	R	D	M	F	E	S	T	K	I	I
Danaus plexippus	K	E	S	C	F	A	S	G	P	G	R	D	M	F	E	S	T	L	I	I
Bombyx mori	K	E	R	C	F	A	S	G	P	G	R	D	M	F	D	S	T	R	I	I
Spodoptera litura	K	E	S	C	F	A	S	G	P	G	R	D	M	F	D	S	T	S	I	I
Manduca sexta	K	E	R	C	F	A	S	G	P	G	R	D	M	F	E	S	T	K	I	I

CDASE	Amino acid position 605 in the CDS of XP_004929418																			
P. acdestis	S	G	H	P	R	N	S	V	R	A	G	R	W	L	A	A	V	E	R	L
P. glacialis	S	G	H	P	R	N	S	A	R	A	G	R	W	L	A	A	V	E	R	L
P. cephalus	S	G	H	P	R	N	S	V	R	A	G	R	W	F	A	A	V	E	R	L
P. epaphus	S	G	H	P	R	N	S	V	R	A	G	R	W	L	A	A	V	E	R	L
P. imperator	S	G	H	P	R	N	S	V	R	A	G	R	W	L	A	A	V	E	R	L
P. jacquemontii	S	G	H	P	R	N	S	V	R	A	G	R	W	L	A	A	V	E	R	L
P. orleans	S	G	H	P	R	N	S	V	R	A	G	R	W	F	A	A	V	E	R	L
P. simo	S	G	H	P	R	N	S	L	R	A	G	R	W	F	A	A	V	E	R	L
Sericinus montelus	S	G	H	P	R	N	S	A	R	H	G	R	W	Y	A	T	V	E	R	L
papilio polytes	S	G	H	P	R	N	S	I	R	H	G	R	W	Y	A	A	V	E	R	L
Papilio xuthus	S	G	H	P	R	N	S	V	S	H	G	R	W	Y	A	A	V	E	R	L
Papilio machaon	S	G	H	P	R	N	S	V	R	H	G	R	W	Y	A	A	V	E	R	L
Pieris rapae	S	G	H	P	R	N	S	I	R	H	G	R	W	Y	S	T	V	E	K	L
Danaus plexippus	S	G	H	P	S	N	S	P	R	H	G	R	W	F	L	S	V	E	R	L
Bombyx mori	S	G	H	P	R	N	S	V	R	H	G	R	W	Y	M	A	V	E	K	L
Spodoptera litura	S	G	H	P	R	N	S	I	R	H	G	R	W	Y	A	T	V	E	R	L
Manduca sexta	S	G	H	P	R	N	S	I	S	H	G	R	W	Y	A	T	V	E	S	L

PGM2	Amino acid position 504 in the CDS of XP_004929437																			
P. acdestis	E	S	G	V	Y	P	K	K	M	G	S	T	G	M	V	Q	L	N	D	F
P. glacialis	A	S	G	V	Y	P	K	K	I	G	C	T	E	M	V	Q	L	N	D	F
P. cephalus	E	S	G	V	Y	P	K	K	M	G	S	T	E	M	V	Q	L	N	D	F
P. epaphus	E	S	G	V	Y	P	K	K	M	G	S	T	E	M	V	Q	L	N	D	F
P. imperator	E	S	G	V	Y	P	K	K	M	G	S	T	G	M	V	Q	L	N	D	F
P. jacquemontii	E	S	G	V	Y	P	K	K	M	G	S	T	E	M	V	Q	L	N	D	F
P. orleans	E	S	G	V	Y	P	K	K	M	G	S	T	E	M	V	Q	L	N	D	F
P. simo	E	S	G	V	Y	P	K	K	M	G	S	T	E	M	V	Q	L	N	D	F
Sericinus montelus	G	S	G	V	Y	P	K	K	V	G	S	C	E	I	E	L	V	N	D	F
papilio polytes	G	S	G	V	Y	P	K	K	V	G	S	C	E	I	V	H	L	N	D	F
Papilio xuthus	G	S	G	V	Y	P	K	K	V	G	S	C	E	I	V	H	M	N	D	F
Papilio machaon	G	S	G	V	Y	P	K	K	V	G	S	C	E	M	V	H	M	N	D	F
Pieris rapae	G	K	G	L	Y	P	K	T	V	G	P	C	E	V	V	F	V	N	D	F
Danaus plexippus	G	P	G	E	Y	P	S	V	V	G	P	C	E	V	T	F	M	N	D	F
Bombyx mori	G	P	G	Q	Y	P	R	R	V	G	S	C	G	V	T	R	V	T	D	F
Spodoptera litura	G	P	G	Q	Y	P	K	K	V	G	D	C	E	I	V	F	V	N	D	F
Manduca sexta	G	P	G	E	Y	P	S	Q	V	G	S	H	E	M	V	F	M	N	D	F
